# Computational insights into shape effects and heat transport enhancement in MHD-free convection of polar ternary hybrid nanofluid around a radiant sphere

**DOI:** 10.1038/s41598-023-47853-8

**Published:** 2024-01-12

**Authors:** Ehab A. El-sayed, Firas A. Alwawi, Fahad Aljuaydi, Mohammed Z. Swalmeh

**Affiliations:** 1 Department of Science and Mathematical Engineering, Faculty of Petroleum and Mining Engineering, Suez University, P.O.BOX 43221 Suez, Egypt; 2 Department of Mathematics, College of Sciences and Humanities in Al-Kharj, Prince Sattam Bin Abdulaziz University, 11942 Al-Kharj, Saudi Arabia; 3 Faculty of Arts and Sciences, Aqaba University of Technology, Aqaba, 77110 Jordan

**Keywords:** Nanoscience and technology, Applied mathematics

## Abstract

The control and management of energy and their associated issues are increasingly recognized as one of mankind’s greatest challenges in the coming years to keep pace with the surge in industrialization and technology. Free convection optimizes the heat transfer processes in energy systems like solar collectors and power plants, reducing energy consumption and increasing system effectiveness. Further, studying and analyzing critical factors like magnetic fields, thermal radiation, and the shape of nanoparticles can assist in the control of fluid motion and improve the efficiency of heat transfer processes in a wide range of real-world applications, such as the power sector, aerospace applications, molten metal, nuclear power, and aeronautical engineering. This study aims to scrutinize the thermal performance of a magneto tri-hybrid polar nanoliquid flowing over a radiative sphere, considering the nanosolids’ shape. The single-phase model is developed to acquire the problems governing equations, and the hybrid linearization spectral collection approach is utilized to approximate the solution. The present findings reveal that blade-shaped nanosolids exhibit the highest thermal conductivity ratio when incorporated into the base fluid, whereas spherical nanosolids exhibit the lowest ratio. Volume fraction and thermal radiation factors have an effective role in raising fluid velocity and thermal performance. The magnetic and microapolar factors significantly suppress fluid velocity and energy transfer. As the volume fraction factor increases, the average percentage improvement in convective heat transfer for Al_2_O_3_ + Cu + MWCNT/kerosene oil compared to Al_2_O_3_ + Cu + graphene/kerosene oil approximately ranges from 0.8 to 2.6%.

## Introduction

Free convection relies on inherent buoyancy forces generated by thermal gradients to drive fluid motion and enable efficient heat transfer. Free convection optimizes the heat transfer processes in energy systems like solar collectors and power plants, reducing energy consumption and increasing system effectiveness. Industrial drying, cooling, and casting operations are made more efficient by free convection, in which molten metal is cast and solidified under better control. The electronics industry takes advantage of free convection by providing an innovative solution for reliable thermal management, effectively regulating the temperature of electronic components. Moreover, free convection is essential to the design and operation of heat exchangers and ventilation systems and contributes to our comprehension of complex environmental and geophysical phenomena^1–4^. Magnetohydrodynamics (MHD) represents an intriguing scientific field that improves the regulation of heat transmission, particularly in liquid metal cooling. MHD enables the use of magnetic fields to control electrically conducting fluids and enhance heat transfer processes. By applying a magnetic field, MHD induces electric currents that interact with the magnetic field, generating Lorentz forces that regulate fluid motion. The effective heat transfer made possible by this controlled fluid motion makes MHD a crucial component of advanced cooling systems for high-temperature applications. In several fields, such as nuclear power, aeronautical engineering, and advanced materials processing, where conventional cooling techniques may have limitations, MHD-based cooling systems have found applications^5,6^. On the other hand, the applications of thermal radiation span numerous sectors and industries. For the power sector, high-temperature operation, and aerospace applications, it is essential. Thermal radiation also plays a critical role in regulating energy transport, especially in polymer manufacturing. Furthermore, in solar energy-based industries, thermal radiation is employed in many applications, like solar energy collectors. Given the vast array of possibilities arising from the applications of free convection as well as the crucial functions of magnetic fields and thermal radiation in the realm of energy transfer, a multitude of numerical studies have delved into this issue. The findings of Sheikholeslami et al.’s^7^ study demonstrated that an improvement in energy transport has a direct relationship with the radiation parameter. Additionally, the study found that the radiation parameter has a positive correlation with the Nusselt number. The results of El-Kabeir et al.^8^ confirmed that as the magnetic force increases, both the skin-friction coefficient and heat transport rate decrease, while a contrasting pattern emerges when it comes to thermal radiation. As thermal radiation intensifies, an increase in the skin-friction coefficient and heat transport rate occurs. In a numerical study performed by Lone et al.^9^, it was revealed that an increase in the magnetic parameter amplifies velocity profiles in the x-direction while simultaneously diminishing them in the z-direction. The study also observed a correlation between an escalation in the magnetic field parameter and a decrease in skin friction, specifically along the x-direction. Additionally, the study found that the Nusselt number experienced a notable increase with an elevation in the thermal radiation parameter. See these intriguing numerical studies^10–13^.

Polar microfluids are frequently characterized as polar, isotropic liquids with no consideration for molecular deformation. Erringen^14^ introduced the micropolar theory in 1966. His simple model, commonly referred to as the “micropolar model”, has gained considerable acceptance and has been adopted to describe the thermal behavior of actual liquids with an internal structure. The flow behavior of liquid crystals, suspension solutions, animal blood, and many other fluids can be characterized by a micropolar fluid model. In recent years, numerous research projects have focused on energy transport characteristics using the micropolar model. Nazar et al.^15,16^ relied on the micropolar model to predict and analyze the thermal behavior of the fluid moving around a spherical object, considering constant wall temperature and heat flux. The findings of their studies indicate that as the micropolar factor increases, both wall temperature and skin friction exhibit a rising trend. Swalmeh et al.^17,18^ extended the studies of Nazar et al. by considering nanofluid issues through the single-phase model. Their findings reveal that the temperature and velocity of Al_2_O3-H_2_O surpass those of Al_2_O_3_-kerosene oil. Furthermore, the energy transport rate of Cu-H2O exhibits a noticeable decline compared to Al_2_O3-H_2_O as the micro-rotation factor escalates. Nabwey et al.^19^ examine the influence of Newtonian heating on magnetohydrodynamic heat transfer induced by natural means of polar nanoliquids across a spherical object. Their validated findings support the notion that the presence of the micropolar factor diminishes skin friction and the energy transport rate. Likewise, their observations indicate that incorporating the Newtonian heating factor enhances both skin friction and the energy transport rate. Other related studies can be found in Refs.^20–22^.

Control and management of energy and their related issues are increasingly recognized as one of mankind’s greatest challenges in the coming years to keep pace with the surge in industrialization and technology^23,24^. One of the innovative proposals is to optimize the performance of energy-transport fluids through the incorporation of metallic and ceramic ultrafine particles into the original fluid to form nanofluid. It all began with the study of Choi and Eastman^25^, who theoretically confirmed that the thermal conductivity of H2O can be markedly enhanced by including copper nanosolids. Afterwards, experimental and numerical studies continued, confirming that the thermal behavior of the reference fluid is significantly affected by nanosolids^26–32^. At present, nanofluids are evidently employed in a wide array of manufacturing and engineering applications, such as solar energy, heat exchangers, and cooling systems^33–37^. Hybrid nanomaterials are a developed class of nanomaterials fabricated from two nanoparticles to obtain the properties of their constituent materials. That is, the main objective of their synthesis is to create a compound with properties that combine thermal and rheological efficiency, as no single nanosolid can possess these properties^38–43^. To acquire features that are more integrated, ternary hybrid nanosolids have been fabricated. Several studies have shown the thermal advantages of these upgraded nanocomposites over the previous class^44–47^. For numerical studies, Mahmood et al.^48^ computationally simulated the unsteady magneto-flow of polymer trihybrid nanofluid around a sphere under the impact of ohmic heating. According to their findings, the magnetic factor and nanosolids concentration enhance heat distribution, while unsteadiness and rotation factors reduce it. In comparison to hybrid and original nanoliquids, tri-hybrid nanoliquids transport energy more rapidly. AlBaidani et al.^49^ conducted a computational simulation to predict the enhancement of fin performance due to the use of tri-hybrid nanosolids, considering the shape factor of nanosolids and free convection. Their key findings indicate that the efficiency of energy performance is significantly influenced by thermal conductivity and free convection. Utilizing magnetic fields and thermal radiation proves to be effective in cooling fins. Tri-hybrid nanosolids enhance the efficiency of fins as opposed to hybrid nanosolids. See also^50,51^.

As a control parameter, the shape of the suspended nanoparticles is among the critical parameters that affect the thermophysical features of nanoliquids. Numerous earlier experimental and numerical publications have highlighted the influence of nanosolid shapes. A numerical study was carried out by Kumar et al.^52^ to explore the flow and thermal features of nanoliquid in a thermally driven cavity. It was found that an increment in the values of the shape factor was accompanied by a significant enhancement in thermal conductivity. Sheikholeslami and Shamlooei^53^ examined the flow of magnetized iron oxide–H2O nanoliquid in a permeable medium, considering the shape factor. Their study showed that platelet-shaped iron oxide nanoparticles achieved the maximum energy transfer rate. Khashi’ie et al.^54^ analyzed the thermal characteristics of Cu–Al2O3/H2O hybrid nanoliquid flow past an EMHD sheet, considering the impact of radiation. Their results supported the idea that as the volume fraction factor values rise, blade-shaped nanosolids exhibit the maximum energy transport rate, while spherical nanosolids exhibit the lowest energy transport rate. Ghobadi and Hassankolaei^55^ carried out a numerical simulation of magnetohydrodynamic hybrid nanoliquid flow across a stretching cylinder. They observed that lamina nanomaterials have a greater effect on the Nusselt number than hexagonal nanomaterials. Shanmugapriya et al.^56^ presented a numerical simulation to explore the efficiency of energy transfer in MHD tri-hybrid nanoliquid on a radiative moving wedge. In their study, they compared the efficiency of energy transfer between different shapes of nanosolids. See^57–59^ for more related studies.

By drawing upon the insights gained from previous studies. This work represents a natural progression from the investigations conducted by Nazar et al.^15,16^ on micropolar fluid flow around a sphere to the more recent advances made by Swalmeh et al.^17,18^ on micropolar nanofluids, along with the expansion that takes into account the micropolar hybrid nanofluid examined by Alkasasbeh et al.^60^. The novelty of the current study is to expand upon these findings by investigating the new problem of a micropolar tri-hybrid nanoliquid moving around a radiative spherical object with the application of a magnetic field. In addition to considering the impacts of a nanosolid’s shape on flow properties and energy transport and highlighting the influences of control factors on some physical groups associated with energy transit. Furthermore, this consideration plays an essential role in numerous physical and engineering applications that rely on heat transmission primarily via electrically conductive fluids. Its applications are considerably obvious, with biomedical applications and flow control around hypersonic and re-entry vehicles. Also, its outcomes could provide new insights into the design and optimization of energy transport systems that use ternary nanoliquids with tailored shapes of the nanosolids. It is anticipated and hoped that the results of this analysis will be beneficial for upcoming academic studies and, additionally, for engineering and practical applications. More precisely, this investigation will demonstrate the following issues:

1. How do the magnetohydrodynamics (MHD) and micropolar tri-hybrid nanoliquid models construct the problem of free convection flow moving around a radiative spherical object?
2. How can a mathematical model for the problem of MHD micropolar tri-hybrid nanoliquid be derived over a radiative spherical object?
3. How does the MHD micropolar tri-hybrid nanoliquid model compare with the published natural heat transfer flow problems?
4. How does the analysis of the numerical outcomes that can be obtained from the effects of MHD micropolar tri-hybrid parameters on the interested engineering physical quantities?
5. How do the heat transfer behaviors of the utilized nanoparticles suspended in the original fluid change under the influence of the studied parameters?

### Thermophysical properties of mono nanoliquid and ternary hybrid nanoliquid

Employing Hamilton and Crosser’s extended Maxwell model^61^, mono nanoliquids’ thermal conductivity, containing similar nanosolids of any shape, is calculated:

1 $$\frac{{k}_{nf}}{{k}_{f}}=\frac{{ k}_{s}+(n-1){ k}_{f}-(n-1) {\chi }_{s} ( { k}_{f}- { k}_{s} )}{{ k}_{s}+(n-1){ k}_{f} + {\chi }_{s} ( { k}_{f}- { k}_{s} )}.$$

The mathematical expression for the viscosity of mono-nanoliquids, which takes into account the shape of nanosolids, is as follows (see^62^):

2 $$\frac{{\mu }_{nf}}{{\mu }_{f}}=1+{C}_{1}{\chi }_{s} +{C}_{2}{\chi }_{s}^{2}.$$

where $n=3/\omega$ is the empirical shape factor, and $\omega$ is the particle’s sphericity, which is defined as the ratio of its spherical surface area to another shape’s surface area, considering both shapes have the same volumes. ${C}_{1}\text{ and }{C}_{2}$ are the vicosity coefficients, which are calculated experimentally at room temperature. The coefficients of viscosity and shape factor of the nanoparticles employed in the current study are listed in Table 1.

**Table 1:** Coefficients of viscosity and shape factor^62,63^.

Shape of nanosolid	Viscosity coefficient		Sphericity $$(\omega )$$	Shape factor ($$n)$$
	${C}_{1}$	${C}_{2}$		
Platelets	37.1	612.6	0.52	5.7
Blades	14.6	123.3	0.36	8.6
Cylinders	13.5	904.4	0.62	4.9
Bricks	1.9	471.4	0.81	3.7
Sphere	2.5	6.2	1	3

The density, specific heat capacity, and thermal expansion of tri-hybrid nanoliquids can be evaluated based on the model presented by Refs.^59,64^ as follows:

3 $$\begin{array}{*{20}l} \rho_{thnf} = \chi_{1} \rho_{1} + \chi_{2} \rho_{2} + \chi_{3} \rho_{3} + \left[ {\left( {1 - \chi_{1} - \chi_{2} - \chi_{3} } \right)\rho_{f} } \right], \hfill \\ (\rho c_{p} )_{thnf} = \chi_{1} (\rho c_{p} )_{1} + \chi_{2} (\rho c_{p} )_{2} + \chi_{3} (\rho c_{p} )_{3} + [\left( {1 - \chi_{1} - \chi_{2} - \chi_{3} } \right)\left( {\rho c_{p} )_{f} } \right], \hfill \\ (\rho \beta )_{thnf} = \chi_{1} (\rho \beta )_{1} + \chi_{2} (\rho \beta )_{2} + \chi_{3} (\rho \beta )_{3} + [\left( {1 - \chi_{1} - \chi_{2} - \chi_{3} } \right)\left( {\rho \beta )_{f} } \right]. \hfill \\ \end{array}$$

Using the interpolation method, the viscosity, thermal conductivity, and electrical conductivity of tri-hybrid nanoliquids can be calculated by employing the following formulas (see^59^):

4 $$\begin{array}{*{20}l} \frac{{\mu_{thnf} }}{{\mu_{f} }} = \frac{{ \mu_{nf1} \chi_{1} + \mu_{nf2} \chi_{2} + \mu_{nf3} \chi_{3} }}{{\chi \mu_{f} }}, \frac{{k_{thnf} }}{{k_{f} }} = \frac{{ k_{nf1} \chi_{1} + k_{nf2} \chi_{2} + k_{nf3} \chi_{3} }}{{\chi k_{f} }}, \hfill \\ \frac{{\sigma_{thnf} }}{{\sigma_{f} }} = \frac{{3\left( {\frac{{\chi_{1} \sigma_{1} + \chi_{2} \sigma_{2} + \chi_{3} \sigma_{3} }}{{\sigma_{f} }} - \chi } \right)}}{{\left( {\frac{{\chi_{1} \sigma_{1} + \chi_{2} \sigma_{2} + \chi_{3} \sigma_{3} }}{{\chi \sigma_{f} }} + 2} \right) - \left( {\frac{{\chi_{1} \sigma_{1} + \chi_{2} \sigma_{2} + \chi_{3} \sigma_{3} }}{{\sigma_{f} }} - \chi } \right)}} . \hfill \\ \end{array}$$

The subscriptions 1 and 2 indicate Al_2_O_3_ and Cu, respectively, while subscription 3 indicates graphene or MWCNT. $\chi ={\chi }_{1}+{\chi }_{2}+{\chi }_{3}$ is the accumulation nanoparticle volume fraction factor. The thermophysical features of the original fluid and the nanosolids utilized in the current study are presented in Table 2.

**Table 2:** Thermophysical features of original fluid and nanosolids^59,65–67^.

Thermo-physical feature	Kerosene Oil (KO)	Al_2_O_3_	Cu	Graphene	MWCNT
$\rho {c}_{p}$(J/kg K)	2090	773	385	790	740
$\rho \beta$×10^−5^ (K^−1^)	22.85	0.85	1.67	− 0.8	44
*ρ* (kg/m^3^)	783	3970	8933	2200	2600
*k* (W/m K)	0.15	40	401	5000	3000
*σ*(s/m)	5 × 10^–11^	1.12 $\times$ 10^5^	3.5 × 10^7^	1 × 10^–7^	1.9 $\times$ 10^–4^
Pr	22.85	…	…	…	…

**Figure 1:**
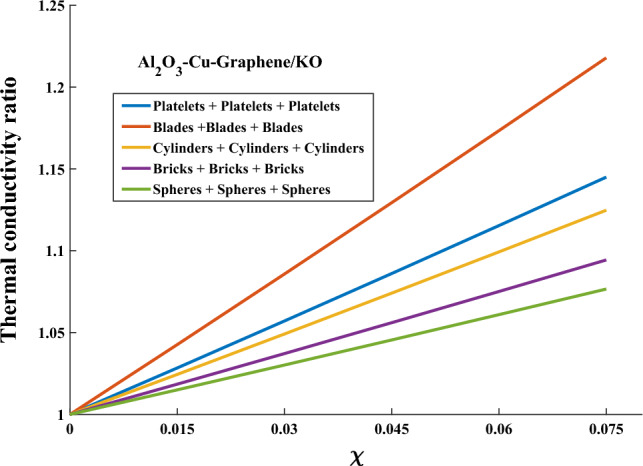
Thermal conductivity versus shape factor.

**Figure 2:**
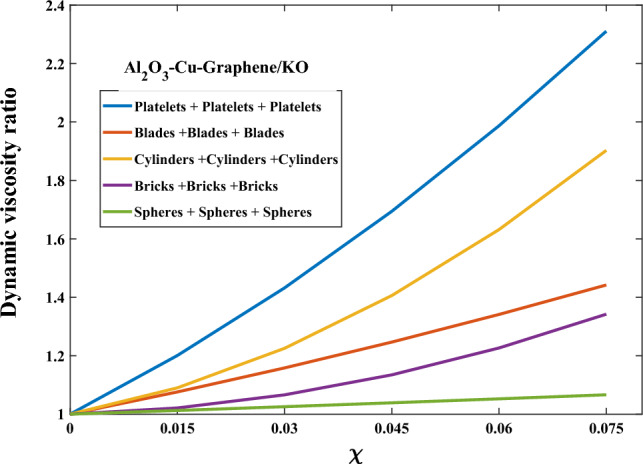
Dynamic viscosity versus shape factor.

Figure 1 presents a visualization of the relationship between the nanosolids shape and the thermal conductivity ratio. The thermal conductivity ratio exhibits an upward trend as the surface area of nanosolids grows. Blade-shaped nanosolids demonstrate the highest thermal conductivity ratio, whilst spherical nanoparticles exhibit the lowest ratio. This confirms that the higher shape factor of nanosolids produces the highest ratio of thermal conductivity. Figure 2 presents a visualization of the relationship between the nanosolids shape and the dynamic viscosity ratio. It is noted that nanosolids with larger elongations (like platelets and cylinders) give kerosene oil the maximum dynamic viscosity ratio due to the structure of these shapes. Therefore, relying on these nanosolid shapes gives the original fluid a higher boiling point, which, of course, enhances its energy-carrying capacity.

### Model’s description

**Figure 3:**
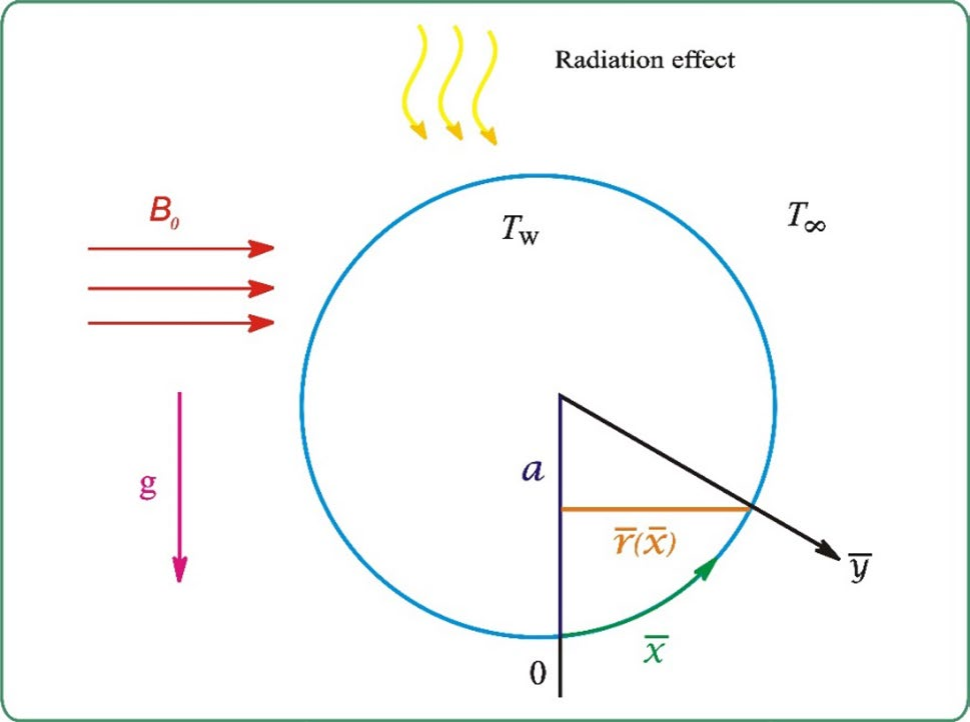
Physical configuration.

Suppose we have a two-dimensional free convection boundary layer flow of kerosene oil containing Al_2_O_3_ + Cu + graphene or Al_2_O_3_ + Cu + MWCNT around a solid sphere of radius *a* considering a thermal radiation effect and a magnetic field of strength *B*_0_. The first-dimensional variable $\overline{x }$ is taken into consideration along the solid sphere’s circumference surface, and the second-dimensional variable $\overline{y }$ is presented perpendicular to it, as offered in Fig. 3. The wall temperature *T*_*w*_ is assumed to be lower than the ambient medium *T*_*∞*_.

In light of the previous considerations and the Boussinesq boundary layer approximations, as well as employing the ternary hybrid nanofluids model, regarding magnetic, thermal radiation, and micropolar impacts, the continuity, momentum, energy, and micropolar equations are developed^20,60,68^:

5 $$\frac{\partial {\overline {r} }{\overline {z}} }{\partial {\overline x} }+\frac{\partial {\overline {r} }{\overline {w}} }{\partial \overline{y} }=0,$$

6 $${\rho }_{thnf} \left(\overline{z } \frac{\partial \overline{z} }{\partial \overline{x} }+\overline{w }\frac{\partial \overline{z} }{\partial \overline{y} }\right)=\left( {\mu }_{thnf}+\kappa \right) \left( \frac{{\partial }^{2}\overline{z} }{\partial {\overline{y} }^{2}} \right) +(\rho {\beta )}_{thnf} {\rho }_{thnf}\mathrm{ g}(T-{T}_{\infty }){\text{sin}}\frac{\overline{x}}{a }+\kappa \left( \frac{\partial \overline{G} }{\partial \overline{y} } \right)-{\sigma }_{thnf}{B}_{0}^{2}\overline{z },$$

7 $$\overline{z} \frac{\partial T}{\partial x }+\overline{w} \frac{\partial T }{\partial \overline{y} }={\alpha }_{thnf}\left(\frac{{\partial }^{2}\overline{T} }{\partial {\overline{y} }^{2}} \right)-\frac{1}{({\rho {c}_{p})}_{thnf}} \frac{1}{({\rho {c}_{p})}_{thnf}} \frac{\partial T}{\partial \overline{y}}\frac{\partial {q }_{r} }{\partial y},$$

8 $${\rho }_{thnf} j\left(\overline{z } \frac{\partial \overline{G} }{\partial \overline{x} }+\overline{w }\frac{\partial \overline{G} }{\partial \overline{y} }\right)={\phi }_{thnf} \left( \frac{{\partial }^{2}\overline{G} }{\partial {\overline{y} }^{2}} \right)-\kappa \left( 2\overline{G }+\frac{\partial \overline{z} }{\partial \overline{y} } \right).$$

It is noted that the vector g (gravity acceleration), that exists in Eq. (6), is implicitly expressed in (*x, y*)-direction, which is defined as two components ${\mathrm{g}}_{x}=\mathrm{g} {\text{si}}{\text{n}}\left(\frac{\overline{x}}{a }\right)$ and ${\mathrm{g}}_{y}=\mathrm{g cos}\left(\frac{\overline{x}}{a }\right)$. Depending on the boundary approximations of the free convection case, the Grashof number *Gr* → ∞, which is equivalent to (1/*Gr*) → 0, the gravity component (${\mathrm{g}}_{y}=\mathrm{g cos}\left(\frac{\overline{x}}{a }\right)$) has been neglected. The constant wall temperature boundary conditions are defined as^20^:

9 $$\begin{array}{*{20}l} \overline{z} = \overline{w} = 0 ,\;T = T_{w} , \;\overline{G} = - \left( {1/2} \right)\frac{{\partial \overline{z}}}{{\partial \overline{y}}},\; {\text{as }}\overline{y} = 0, \hfill \\ \overline{w} \to 0, \;T \to T_{\infty } , \;\overline{G} \to 0, \;{\text{as }}\overline{y}{ } \to \infty , \hfill \\ \end{array}$$

where ${q}_{r}=-\frac{4{\sigma }^{*}}{3{k}^{*}}{\left(\frac{\partial {T}^{4}}{\partial \overline{y} }\right)}_{\overline{y }= 0}$, and ${T}^{4}\cong 4{T}_{\infty }^{3}T-3{T}_{\infty }^{4}$. ${\sigma }^{*},\,and\, {k}^{*}$ are the Stefan–Boltzmann and mean absorption coefficients, respectively. The appropriate non-dimensional variables are^16^:

10 $$\begin{gathered} x = \frac{{\overline{x}}}{a}, y = Gr^{1/4} a^{ - 1} \overline{y,} \,\,\,\,\,\,r\left( x \right) = \overline{r}\left( {\overline{x}} \right)/a, \theta = \frac{{\left( {T - T_{\infty } } \right)}}{{{ } T_{w} - { } T_{\infty } }}, \hfill \\ z = \frac{a}{{v_{f} }}Gr^{ - 1/2} \overline{z}, \,\,\,\,\,\,\,w = \frac{a}{{v_{f} }}Gr^{ - 1/4} \overline{w}, \hfill \\ \end{gathered}$$

where $j= {a}^{2}/G{r}^{1/2}$ is micro-inertia density, *Gr* =$g{a}^{3}(T-{T}_{\infty })(\rho {\beta )}_{f}/{{v}_{f}}^{2}$ is the Grashof number, and $\overline{r }\left(\overline{x }\right)=a \mathrm{sin}\left(\frac{\overline{x}}{a }\right)$ is the radial distance. Substituting the variables (10) into Eqs. (5)–(9), yields the following nondimensional equations:

11 $$\frac{\partial rz}{\partial x}+\frac{\partial rw}{\partial y}=0,$$

12 $$z \frac{\partial z}{\partial x}+w\frac{\partial z}{\partial y}=\frac{{\rho }_{f}}{{\rho }_{thnf}}\left( \frac{{\mu }_{thnf}}{{\mu }_{f}}+K \right)\frac{{\partial }^{2}z}{\partial {y}^{2}}+\frac{({\rho \beta )}_{thnf}}{({\rho \beta )}_{f}}\theta {\text{sin}}x+\frac{{\rho }_{f}}{{\rho }_{thnf}}K \frac{\partial G}{\partial y}-\frac{{\rho }_{f}}{{\rho }_{thnf}} \frac{{\sigma }_{thnf}}{{\sigma }_{f}}\mathrm{M}z,$$

13 $$z\frac{\partial \theta }{\partial x} +w \frac{\partial \theta }{\partial y}=\frac{1}{Pr}\frac{({\rho {c}_{p})}_{f}}{({\rho {c}_{p})}_{thnf}}\left(\frac{{k}_{thnf}}{{k}_{f}}+\frac{4}{3}L\right)\frac{{\partial }^{2}\theta }{\partial {y}^{2}} ,$$

14 $$z\frac{\partial G}{\partial x}+w\frac{\partial G}{\partial y}=\frac{{\rho }_{f}}{{\rho }_{thnf}} \left(\frac{{ \mu }_{thnf}}{{\mu }_{f}}+\frac{K}{2 }\right) \frac{{\partial }^{2}G}{\partial {y}^{2}}-\frac{{\rho }_{f}}{{\rho }_{thnf}}K\left( 2G+\frac{\partial z}{\partial y} \right).$$

where $K=\kappa /{\mu }_{f}$, $L={4\sigma }^{*}{T}_{\infty }^{3}/{k}_{f}4{k}^{*}$, $\mathrm{M}={{\sigma }_{f} a}^{2}{B}_{0}^{2}{Gr}^{-1/2}/{\rho }_{f}{v}_{f}$, and $\mathrm{Pr}={(v}_{f}/{\alpha }_{f})$ are the micropolar factor, radiation factor, magnetic factor, and Prandtl number, respectively.

The mathematical model (11)–(14) can be reduced using the following non-similar transformation (stream function $\psi$):

15 $$z=\frac{1}{r}\frac{\partial \psi }{\partial y},\mathrm{ \,and\, }w=-\frac{1}{r}\frac{\partial \psi }{\partial x},$$

where $$\psi =x r(x)f\left( x, y \right),\theta =\theta \left( x,y \right),G=x h\left( x, y\right)$$.

Utilizing the non-similar transformations (15) and using Eqs. (1)–(4) yields:

16 $$\frac{{\rho }_{f}}{{\rho }_{thnf}}\left( \frac{{\mu }_{thnf}}{{\mu }_{f}}+K \right)\frac{{\partial }^{3}f}{\partial {y}^{3}}+\left(1+x\mathrm{cot}x\right)f\frac{{\partial }^{2}f}{\partial {y}^{2}}-{\left(\frac{\partial f}{\partial y}\right)}^{2}+\frac{({\rho \beta )}_{thnf}}{({\rho \beta )}_{f}}\theta \frac{{\text{sin}}x}{x}+\frac{{\rho }_{f}}{{\rho }_{thnf}}K \frac{\partial h}{\partial y}-\frac{{\rho }_{f}}{{\rho }_{thnf}} \frac{{\sigma }_{thnf}}{{\sigma }_{f}}M\,\frac{\partial f}{\partial y}= x\,\left(\frac{\partial f}{\partial y}\frac{{\partial }^{2}f}{\partial x\partial y}- \,\frac{\partial f}{\partial x}\frac{{\partial }^{2}f}{\partial {y}^{2}}\right),$$

17 $$\frac{1}{Pr}\frac{({\rho {c}_{p})}_{f}}{({\rho {c}_{p})}_{thnf}}\left(\frac{{k}_{thnf}}{{k}_{f}}+\frac{4}{3}L\right)\frac{{\partial }^{2}\theta }{\partial {y}^{2}} +\left(1+x{\text{cot}}\,x\right)f\frac{\partial \theta }{ \partial y}=x\left(\frac{\partial f}{\partial y}\frac{\partial \theta }{\partial x}- \frac{\partial f}{\partial x}\frac{\partial \theta }{\partial y}\right),$$

18 $$\frac{{\rho }_{f}}{{\rho }_{thnf}}\left(\frac{{ \mu }_{thnf}}{{\mu }_{f}}+\frac{K}{2 }\right) \frac{{\partial }^{2}h}{\partial {y}^{2}}+\left(1+x\mathrm{cot}x\right)f\frac{\partial h}{\partial y}-\frac{\partial f}{\partial y}h-\frac{{\rho }_{f}}{{\rho }_{thnf}}K\left( 2h+\frac{{\partial }^{2}f}{\partial {y}^{2}} \right)=x\left(\frac{\partial f}{\partial y} \frac{\partial h}{\partial x}- \frac{\partial f}{\partial x}\frac{\partial h}{\partial y}\right),$$

subject to:

19 $$\begin{array}{l}f=\frac{\partial f}{\partial y}=0, \theta =1 , h=-(1/2) \frac{{\partial }^{2}f}{\partial {y}^{2}},\mathrm{ as\,}y=0,\\ \frac{\partial f}{\partial y}\to 0, \theta \to 0, h\to 0,\mathrm{ as}\,y \to \infty .\end{array}$$

The skin friction *C*_*f*_ and the Nusselt number *Nu* are (see^16,20^):

20 $$Nu=\left(\frac{a{q}_{w}}{{k}_{f}({T}_{w}-{T}_{\infty})}\right), {C}_{f}=\frac{{\tau }_{w}}{{{ U}_{\infty }^{2} \rho }_{f}},$$

where

21 $$\begin{array}{*{20}l} q_{w} = - k_{thnf} \left( {\frac{\partial T}{{\partial y^{*} }}} \right)_{{\overline{y} = \,0}} \,{ + }\left( {q_{r} } \right)_{{\overline{y} = \,0}} , \hfill \\ U_{\infty }^{2} = \frac{{ Gr v_{f}^{2} }}{{a^{2} }}, \tau_{w} = { }\left( {\mu_{thnf} + \frac{\kappa }{2}} \right)\left( {\frac{\partial z}{{\partial y^{*} }}} \right)_{{\overline{y} = \,0}} . \hfill \\ \end{array}$$

Using the Eqs. (21), (10), and (15), we get:

22 $${Gr}^{-1/4}Nu=-\left( \frac{{k}_{thnf}}{{k}_{f}}+\frac{4}{3}L\right)\frac{\partial \theta }{\partial y}\left(x,0\right), {Gr}^{1/4}{C}_{f}=\left(\frac{{\mu }_{thnf }}{{\mu }_{f}}+\frac{\mathrm{K}}{2}\right) x\frac{{\partial }^{2}f}{\partial {y}^{2}} \left(x,0\right).$$

### Hybrid linearization spectral collection method

In this section, the hybrid linearization spectral collocation technique (HLSC) combined Newton’s linearization method (NLM) with Chebyshev spectral collocation method (CSCM) in $\mathrm{y}$-direction. Firstly, NLM is utilized to linearize and decouple the nonlinear PDEs which are solved using Chebyshev spectral method (see^69–71^).

System (16)–(19) can be written as:

23 $${f}^{\prime}=g,$$

24 $${A}_{1}{\mathrm{g}}^{{\prime}{\prime}}+ \left(1+x\mathrm{cot}x\right)f^{\prime}{\mathrm{g}}-{\left(\mathrm{g}\right)}^{2} +{A}_{2}\theta \frac{{\text{sin}}x}{x}+{A}_{3} \frac{\partial h}{\partial y}+{A}_{4}=x\left(\mathrm{g}\frac{\partial \mathrm{g}}{\partial x}-\frac{\partial f}{\partial x}{\mathrm{g}}^{\prime}\right),$$

25 $${A}_{5}{\theta }^{{\prime}{\prime}}+\left(1+x{\text{cot}} x\right)f^{\prime}{\theta }=x\left(\mathrm{g}\frac{\partial \theta }{\partial x}- \frac{\partial f}{\partial x}{\theta }^{\prime}\right),$$

26 $${A}_{6} {h}^{{\prime}{\prime}}+\left(1+x\mathrm{cot}x\right)f^{\prime}{h}-\mathrm{g}h+{A}_{7}\left( 2h+{\mathrm{g}}^{\prime} \right)=x\left(\mathrm{g} \frac{\partial h}{\partial x}- \frac{\partial f}{\partial x}{h}^{\prime}\right),$$

with the boundary conditions:

27 $$\left.\begin{array}{c}y=0: f=0, \mathrm{g}=0, \,\,\,\,\,\,\,\theta =1 ,\,\,\,\,\,\,\,h=-(1/2) {\mathrm{g}}^{\prime}\\ y\to \infty : \,\,\,\,\,\,\,\mathrm{g}\to 0,\,\,\,\,\,\,\,\theta \to 0, h\to 0\end{array}\right\} ,$$

where $${A}_{1}=\frac{{\rho }_{f}}{{\rho }_{thnf}}\left( \frac{{\mu }_{thnf}}{{\mu }_{f}}+K\right)$$, $${A}_{2}=({\chi }_{1}({\rho \beta )}_{1}/({\rho \beta )}_{f}+{\chi }_{2}({\rho \beta )}_{2}/({\rho \beta )}_{f}+{\chi }_{3}({\rho \beta )}_{3}/({\rho \beta )}_{f}+[(1-\chi )])$$, $${A}_{3}=\frac{{\rho }_{f}}{{\rho }_{thnf}}K,$$ $${A}_{4}= \frac{-{\rho }_{f}}{{\rho }_{thnf}} \frac{{\sigma }_{thnf}}{{\sigma }_{f}}M$$, $${A}_{6}=\frac{{\rho }_{f}}{{\rho }_{thnf}} \left(\frac{{ \mu }_{thnf}}{{\mu }_{f}}+\frac{K}{2}\right)$$, $${A}_{7}=\frac{{-\rho }_{f}}{{\rho }_{thnf}}K,$$ $${A}_{5}=\frac{1}{Pr}\frac{1}{{(\chi }_{1}({\rho {c}_{p})}_{1}/({\rho {c}_{p})}_{f}+{\chi }_{2}({\rho {c}_{p})}_{2}/({\rho {c}_{p})}_{f}+{\chi }_{3}({\rho {c}_{p})}_{3}/({\rho {c}_{p})}_{f}+\left[\left(1-\chi \right)\right]}\left(\frac{{k}_{thnf}}{{k}_{f}}+\frac{4}{3}L\right)$$.

Applying, NLM^72^ to the nonlinear PDEs (23)–(27) results in:

28 $${f}^{\prime}_{n+1}={\mathrm{g}}_{n },$$

29 $${{{\mathrm{A}}_{1}\mathrm{g}}_{n+1}^{{\prime}{\prime}}+\mathrm{a}1}_{n}{\mathrm{g}}^{\prime}_{n+1}+{a2}_{n}{\mathrm{g}}_{n+1}={a3}_{n}+{a4}_{n}\frac{\partial {\mathrm{g}}_{n+1}}{\partial x},$$

30 $${{{\mathrm{A}}_{5}\theta }_{n+1}^{{\prime}{\prime}}+b1}_{n}{\theta^{\prime} }_{n+1}+{b2}_{n}{\theta }_{n+1}={b3}_{n}+{b4}_{n}\frac{\partial {\theta }_{n+1}}{\partial x},$$

31 $${{{\mathrm{A}}_{6}h}_{n+1}^{{\prime}{\prime}}+c1}_{n}{h^{\prime}}_{n+1}+{c2}_{n}{h}_{n+1}={c3}_{n}+{c4}_{n}\frac{\partial {h}_{n+1}}{\partial x},$$

where $n=\mathrm{0,1},2,\dots ,$ and BCs are:

32 $$\left.\begin{array}{c}{f}_{n+1}\left(x,0\right)={g}_{n+1}\left(x,0\right)=0, {\theta }_{n+1}\left(x,0\right)=1, {h}_{n+1}\left(x,0\right)=-\left(1/2\right){g^{\prime}}_{n+1}\left(x,0\right)\\ {g}_{n+1}\left(x,{y}_{\infty }\right)\to 0, {\theta }_{n+1}\left({x,y}_{\infty }\right)\to 0, {h}_{n+1}\left({x,y}_{\infty }\right)\to 0 \end{array}\right\},$$

where the coefficients in the system (28)–(31) are defined as:

33 $$\left.\begin{array}{c}{\mathrm{a}1}_{\mathrm{n}}=\left(1+{x}_{k}\mathrm{cot}{x}_{k}\right){f}_{n}+{x}_{k}\frac{\partial {f}_{n}}{\partial x}, {\mathrm{a}2}_{\mathrm{n}}=-2{\mathrm{g}}_{n}+{A}_{4 }-{x}_{k}\frac{\partial {\mathrm{g}}_{n}}{\partial x}, {\mathrm{a}4}_{\mathrm{n}}={x}_{k}{\mathrm{g}}_{n}\\ {\mathrm{a}3}_{\mathrm{n}}=-\left({\mathrm{g}}_{\mathrm{n}}^{2}+{A}_{2}{\uptheta }_{n}\frac{\mathrm{sin}{x}_{k}}{{x}_{k}}+{A}_{3}{h^{\prime}}_{n}+{x}_{k}{\mathrm{g}}_{n}\frac{\partial {\mathrm{g}}_{n}}{\partial x}\right), {b1}_{n}={\mathrm{a}1}_{\mathrm{n}}, {b2}_{n}=0 , {\mathrm{b}3}_{\mathrm{n}}=0\\ {{\mathrm{b}4}_{\mathrm{n}}=\mathrm{a}4}_{\mathrm{n}}{, c1}_{n}={\mathrm{a}1}_{\mathrm{n}}, {c2}_{n}=2{A}_{7}-\mathrm{g}, {c3}_{n}={-\mathrm{g^{\prime}}}{A}_{7}, {\mathrm{ c}4}_{\mathrm{n}}={\mathrm{a}4}_{\mathrm{n}}\end{array}\right\},$$

where

34 $${f}_{n}={f}_{n}\left({x}_{k}{,y}_{j}\right), {\mathrm{g}}_{\mathrm{n}}={\mathrm{g}}_{n}\left({x}_{k}{,y}_{j}\right), {\theta }_{n}={\theta }_{n}\left({x}_{k}{,y}_{j}\right), {\mathrm{h}}_{\mathrm{n}}={\mathrm{h}}_{n}\left({x}_{k}{,y}_{j}\right).$$

In Eqs. (28)–(33) are a decoupled linear PDEs system where the terms subscripted by n are known from the previous iteration level, and the terms subscripted by n + 1 are the current approximation. The linearized system (28)–(33) is solved by CSCM in $y$-direction and the two-point implicit finite difference approach in $x$-direction, where Chebyshev polynomials are typically selected with their corresponding collocation points in the interval [− 1*,*1]. The points $\left({x}_{k}, {y}_{j}\right)$ are (see^73–75^):

35 $${x}_{k}=k{\Delta x}_{k}\mathrm{\,and\,}{y}_{j}=\frac{1}{2}\left[1-\mathrm{cos}\frac{j\pi }{{N}_{{\overline{y} }_{\infty }}}\right]{\overline{y} }_{\infty }, k=\mathrm{0,1},\dots , {N}_{x} , j=\mathrm{0,1},\dots , {N}_{{\overline{y} }_{\infty }},$$

where ${\Delta x}_{k}$ is the step-size in $\mathrm{x}$-direction, ${\overline{\mathrm{y}} }_{\infty }$ is the initial approximation of ${y}_{\infty }$, ${N}_{x}\, and\, { N}_{{\overline{y} }_{\infty }}$ are the number of subintervals in $x$ and $y$ directions, respectively. The following linear differential transformation is applied to convert the system (28)–(33) into algebraic systems of equations in the y-direction:

36 $$\left.\begin{array}{c}{F}_{n+1}^{\left(m\right)}\left({x}_{k}{,y}_{j}\right)={D}^{m}{F}_{n+1}\left({x}_{k}{,y}_{j}\right)\\ {G}_{n+1}^{\left(m\right)}\left({x}_{k}{,y}_{j}\right)={D}^{m}{G}_{n+1}\left({x}_{k}{,y}_{j}\right)\\ {\Theta }_{n+1}^{\left(m\right)}\left({x}_{k}{,y}_{j}\right)={D}^{m}{\Theta }_{n+1}\left({x}_{k}{,y}_{j}\right)\\ {H}_{n+1}^{\left(m\right)}\left({x}_{k}{,y}_{j}\right)={D}^{m}{H}_{n+1}\left({x}_{k}{,y}_{j}\right)\end{array}\right\} k=0:{N}_{x}, j=0:{N}_{{\overline{y} }_{\infty }}, m=\mathrm{1,2},$$

where ${\mathrm{D}}^{1},\mathrm{\, and\, }{\mathrm{D}}^{2}$ are the 1st and 2nd derivatives Chebyshev differentiation matrices, respectively, given in Refs.^73–75^, that are converted into our entire physical domain $[0,{\overline{\mathrm{y}} }_{\infty } ]$, ${F}_{n+1}=\left[{f}_{n+1}\left({x}_{k}{,y}_{j}\right)\right]$, ${G}_{n+1}=\left[{\mathrm{g}}_{n+1}\left({x}_{k}{,y}_{j}\right)\right]$, ${\Theta }_{n+1}=\left[{\theta }_{n+1}\left({x}_{k}{,y}_{j}\right)\right]$ and ${H}_{n+1}=\left[{h}_{n+1}\left({x}_{k}{,y}_{j}\right)\right]$. While ${F}_{n+1}^{\left(m\right)}$, ${G}_{n+1}^{\left(m\right)}$, ${\Theta }_{n+1}^{\left(m\right)}$ and ${H}_{n+1}^{\left(m\right)}$ are the derivative vectors of $\left[{f}_{n+1}^{\left(m\right)}\left({x}_{k}{,y}_{j}\right)\right]$, $\left[{\mathrm{g}}_{n+1}^{\left(m\right)}\left({x}_{k}{,y}_{j}\right)\right]$, $\left[{\theta }_{n+1}^{\left(m\right)}\left({x}_{k}{,y}_{j}\right)\right]$ and $\left[{h}_{n+1}^{\left(m\right)}\left({x}_{k}{,y}_{j}\right)\right]$, respectively. In the $x$-direction, the two-point backward difference scheme looks like:

37 $${\left.\frac{\partial\Gamma }{\partial x}\right|}_{{n+1 (x}_{k}{,y}_{j)}}=\frac{{\Gamma }_{n+1}\left({x}_{k}{,y}_{j}\right)-{\Gamma }_{n+1}\left({x}_{k-1}{,y}_{j}\right)}{\Delta x}, k=1:{N}_{x}, j=0:{N}_{{\overline{y} }_{\infty }}.$$

The first order derivatives with respect to $x$ are discretized using $\Gamma \left({x}_{k}{,y}_{j}\right)=\mathrm{g}\left({x}_{k}{,y}_{j}\right)$ or $\theta \left({x}_{k}{,y}_{j}\right),$ or $h\left({x}_{k}{,y}_{j}\right)$. The following system for each line ${x}_{k}$ is obtained by applying CSCM to Eqs. (28)–(33):

38 $$\left.\begin{array}{c}\begin{array}{c}{\mathrm{D}}^{1}{F}_{n+1}\left({x}_{k}{,y}_{j}\right)={\mathrm{G}}_{n }\left({x}_{k}{,y}_{j}\right)\\ \left[{\mathrm{A}}_{1}{{D}^{2}{G}_{n+1}+\mathrm{a}1}_{\mathrm{n}}{\mathrm{D}}^{1}{G}_{n+1}+{\mathrm{a}2}_{\mathrm{n}}{\mathrm{G}}_{\mathrm{n}+1}\right]\left({x}_{k}{,y}_{j}\right)={\mathrm{a}3}_{\mathrm{n}}+{a4}_{n}\frac{{\mathrm{G}}_{\mathrm{n}+1}\left({x}_{k}{,y}_{j}\right)-{\mathrm{G}}_{\mathrm{n}+1}\left({x}_{k-1}{,y}_{j}\right)}{\Delta x}\\ \left[{\mathrm{A}}_{5}{{D}^{2}{\Theta }_{n+1}+\mathrm{b}1}_{\mathrm{n}}{\mathrm{D}}^{1}{\Theta }_{n+1}+{\mathrm{b}2}_{\mathrm{n}}{\Theta }_{n+1}\right]\left({x}_{k}{,y}_{j}\right)={\mathrm{b}3}_{\mathrm{n}}+{b4}_{n}\frac{{\Theta }_{\mathrm{n}+1}\left({x}_{k}{,y}_{j}\right)-{\Theta }_{\mathrm{n}+1}\left({x}_{k-1}{,y}_{j}\right)}{\Delta x}\\ \left[{\mathrm{A}}_{6}{{D}^{2}{\mathrm{H}}_{n+1}+\mathrm{c}1}_{\mathrm{n}}{\mathrm{D}}^{1}{\mathrm{H}}_{n+1}+{\mathrm{c}2}_{\mathrm{n}}{\mathrm{H}}_{\mathrm{n}+1}\right]\left({x}_{k}{,y}_{j}\right)={\mathrm{c}3}_{\mathrm{n}}+{c4}_{n} \frac{{\mathrm{H}}_{\mathrm{n}+1}\left({x}_{k}{,y}_{j}\right)-{\mathrm{H}}_{\mathrm{n}+1}\left({x}_{k-1}{,y}_{j}\right)}{\Delta x} \end{array}\end{array}\right\} ,$$

$$k=1:{N}_{x}, j=0:{N}_{{\overline{y} }_{\infty }}.$$

Subject to boundary conditions:

39 $$\left.\begin{array}{c}{F}_{n+1}\left({x}_{k},0\right)={\mathrm{G}}_{n+1}\left({x}_{k},0\right)=0 , {\Theta }_{n+1}\left({x}_{k},0\right)=1, { H}_{n+1}\left({x}_{k},0\right)=-\left({\mathrm{D}}^{1}{G}_{n+1}\left({x}_{k},0\right)/2\right), \\ {\mathrm{ G}}_{n+1}\left({x}_{k}{,N}_{{\overline{y} }_{\infty }}\right)\to 0, {\Theta }_{n+1}\left({x}_{k}{,N}_{{\overline{y} }_{\infty }}\right)\to 0, {\mathrm{ H}}_{n+1}\left({x}_{k}{,N}_{{\overline{y} }_{\infty }}\right)\to 0\end{array}\right\}$$

Here, Eq. (38)s coefficients are the coefficients stated in system (33) expressed in vectors form. The system (38) and (39) is solved iteratively at ${x}_{k}$, $k=1:{N}_{x}$. The above Eqs. (38) and (39), at the point (${x}_{0}\approx 0$), $k=0$, $j=0:{N}_{{\overline{y} }_{\infty }}$ can be determined as the following:

40 $$\left.\begin{array}{c}\begin{array}{c}{\mathrm{D}}^{1}{F}_{n+1}\left({x}_{0}{,y}_{j}\right)={\mathrm{G}}_{n }\left({x}_{0}{,y}_{j}\right)\\ \left[{\mathrm{A}}_{1}{{D}^{2}{G}_{n+1}+\mathrm{a}1}_{\mathrm{n}}{\mathrm{D}}^{1}{G}_{n+1}+{\mathrm{a}2}_{\mathrm{n}}{\mathrm{G}}_{\mathrm{n}+1}\right]\left({x}_{0}{,y}_{j}\right)={\mathrm{a}3}_{\mathrm{n}}\\ \left[{\mathrm{A}}_{5}{{D}^{2}{\Theta }_{n+1}+\mathrm{b}1}_{\mathrm{n}}{\mathrm{D}}^{1}{\Theta }_{n+1}\left({x}_{0}{,y}_{j}\right)+{\mathrm{b}2}_{\mathrm{n}}{\Theta }_{n+1}\right]\left({x}_{0}{,y}_{j}\right)={\mathrm{b}3}_{\mathrm{n}}\\ \left[{\mathrm{A}}_{6}{{D}^{2}{\mathrm{H}}_{n+1}+\mathrm{c}1}_{\mathrm{n}}{\mathrm{D}}^{1}{\mathrm{H}}_{n+1}+{\mathrm{c}2}_{\mathrm{n}}{\mathrm{H}}_{\mathrm{n}+1}\right]\left({x}_{0}{,y}_{j}\right)={\mathrm{c}3}_{\mathrm{n}} \end{array}\end{array}\right\}.$$

Subject to boundary conditions

41 $$\left.\begin{array}{c}{F}_{n+1}\left(0,0\right)={\mathrm{G}}_{n+1}\left(0,0\right)=0 , {\Theta }_{n+1}\left(0,0\right)=1, {, H}_{n+1}\left(0,0\right)=-\left({\mathrm{D}}^{1}{G}_{n+1}\left(0,0\right)/2\right) \\ {\mathrm{ G}}_{n+1}\left(0{,N}_{{\overline{y} }_{\infty }}\right)\to 0, {\Theta }_{n+1}\left(0{,N}_{{\overline{y} }_{\infty }}\right)\to 0, {\mathrm{ H}}_{n+1}\left(0{,N}_{{\overline{y} }_{\infty }}\right)\to 0\end{array}\right\} ,$$

where the coefficients in system (40) are defined as:

42 $$\left.\begin{array}{c}{\mathrm{a}1}_{\mathrm{n}}=2{f}_{n}, {\mathrm{a}2}_{\mathrm{n}}=-2{\mathrm{g}}_{n}+{A}_{4 }, {\mathrm{a}4}_{\mathrm{n}}=0, {\mathrm{a}3}_{\mathrm{n}}=-\left({\mathrm{g}}_{\mathrm{n}}^{2}+{A}_{2}{\uptheta }_{n}+{A}_{3}{h}^{\prime}_{n}\right), {b1}_{n}={\mathrm{a}1}_{\mathrm{n}}\\ {b2}_{n}={\mathrm{b}3}_{\mathrm{n}}={\mathrm{b}4}_{\mathrm{n}}=0{, c1}_{n}={\mathrm{a}1}_{\mathrm{n}}, {c2}_{n}=2{A}_{7}-\mathrm{g}, {c3}_{n}={-\mathrm{g}}^{\prime}{A}_{7} , {\mathrm{c}4}_{\mathrm{n}}=0\end{array}\right\}.$$

The iterative process of each the systems (40), (41) and (38), (39) is terminated if there is a difference of less than ${10}^{-6}$ between the outcomes of two successive iterations. Subject to the BCs (32) hence, suitable initial approximations are:

43 $$\left.\begin{array}{c}{F}_{0}\left(0{,y}_{j}\right)=-1+{e}^{-{y}_{j}^{2}}, {\mathrm{G}}_{0}\left(0{,y}_{j}\right)=-2{{,y}_{j}e}^{-{y}_{j}^{2}}\\ {\Theta }_{0}\left(0{,y}_{j}\right)={e}^{-{y}_{j}}, and {H}_{0}\left(0{,y}_{j}\right)=\left(1-2{y}_{j}^{2}\right){e}^{-{y}_{j}^{2}}\end{array}\right\} , j=0:{N}_{{\overline{y} }_{\infty }}.$$

Once the MATLAB program has been established, we need to set the convergence standards. This requires identifying some important calculations: the proper step sizes (∆*x* and ∆*y*) and the boundary layer thickness (*y* = ∞). In this study, *y* = ∞ should be set between 3 and 8 to achieve boundary layer convergence. Once we choose the appropriate value of *y* = ∞, we can determine the step sizes: ∆*x* = 0.005 and *y* = 0.02. These step sizes will give us valid approximate numerical results that agree with previous research. To ensure the precision of the current technique, the present outcomes are compared with the results provided by Nazar et al.^16^ when the factors $K, \mathrm{L}, M, {\chi }_{1}, { \chi }_{2},\mathrm{ \,and\, }{\chi }_{3}$ where set to zero. See Table 3.

**Table 3:** Comparison of Nazar et al.^16^ results with the current results for ${Gr}^{-1/4}Nu$.

*x*	Nazar et al. ^16^ results Pr = 7	Present results Pr = 7
0°	0.9595	0.9575
10°	0.9572	0.9553
20°	0.9506	0.9489
30°	0.9397	0.9382
40°	0.9243	0.9230
50°	0.9045	0.9034
60°	0.8801	0.8793
70°	0.8510	0.8503
80°	0.8168	0.8163
90°	0.7792	0.7770
100°	–	0.7321
110°	–	0.6808
120°	–	0.6226

## Results and discussion

**Figure 4:**
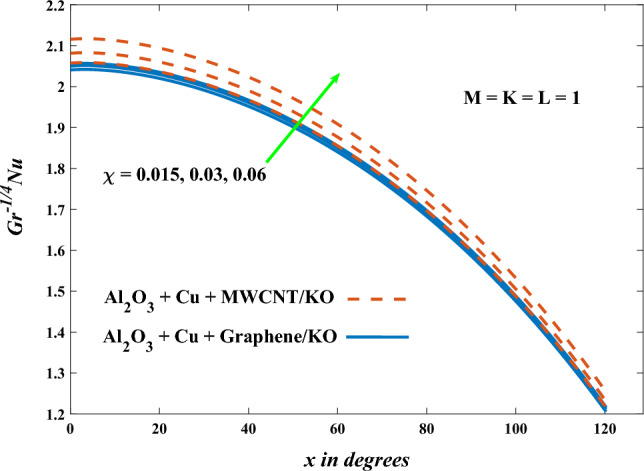
Nusselt number vs. volume fraction factor.

**Figure 5:**
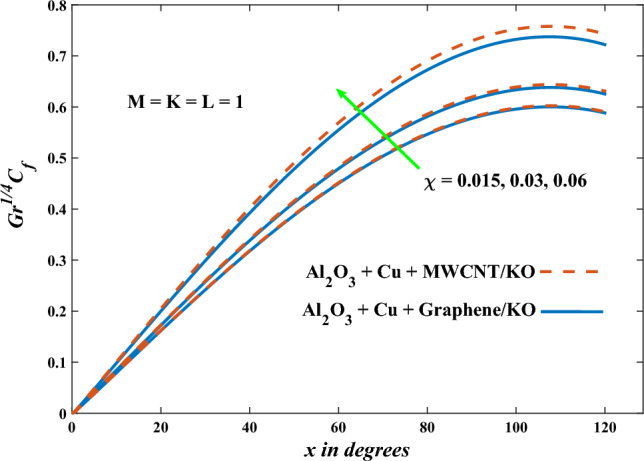
Skin friction vs. volume fraction factor.

**Figure 6:**
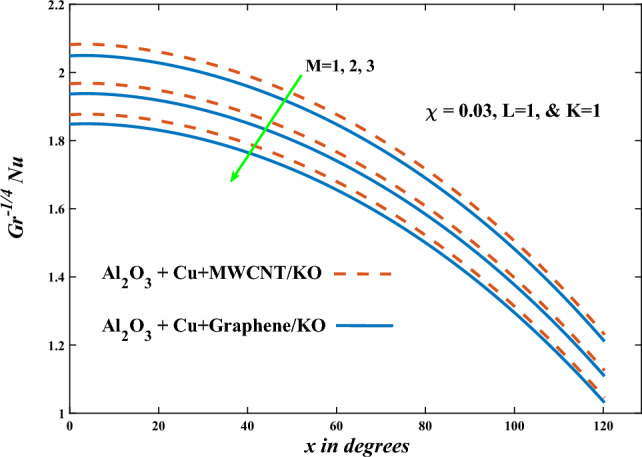
Nusselt number vs magnetic factor.

**Figure 7:**
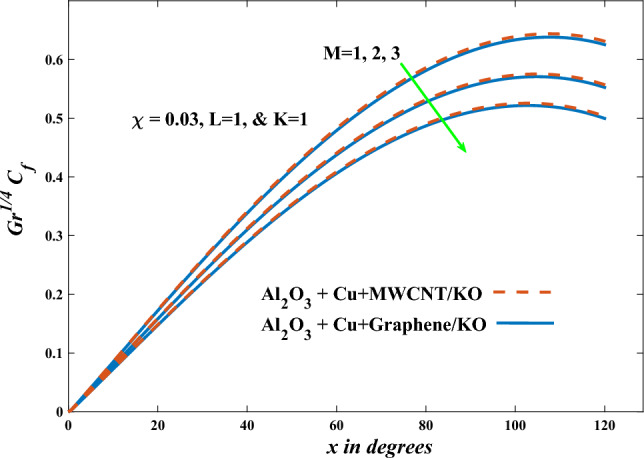
Skin friction vs magnetic factor.

**Figure 8:**
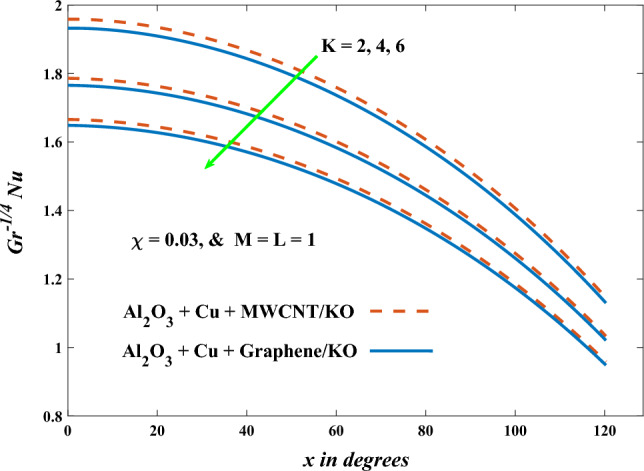
Nusselt number vs micropolar factor.

**Figure 9:**
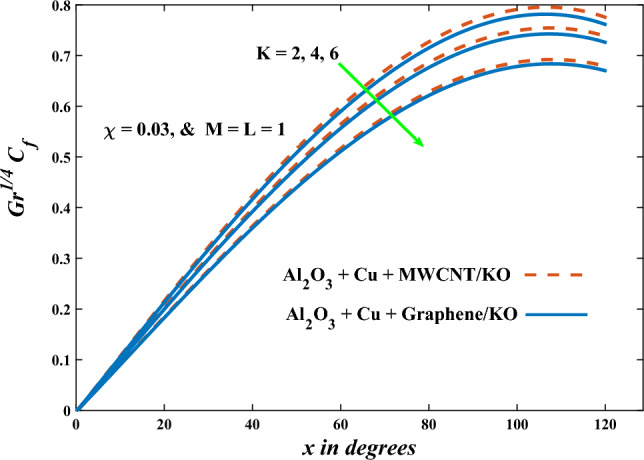
Skin friction vs micropolar factor.

**Figure 10:**
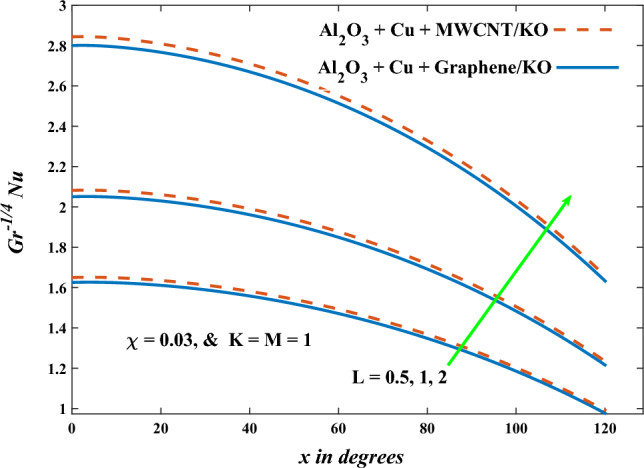
Nusselt number vs radiation factor.

**Figure 11:**
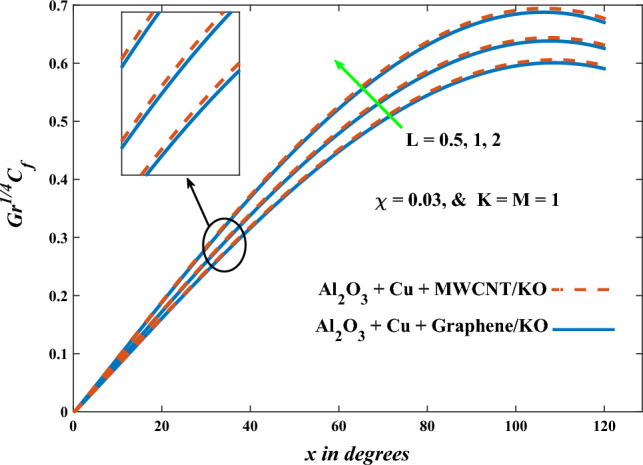
Skin friction vs radiation factor.

**Figure 12:**
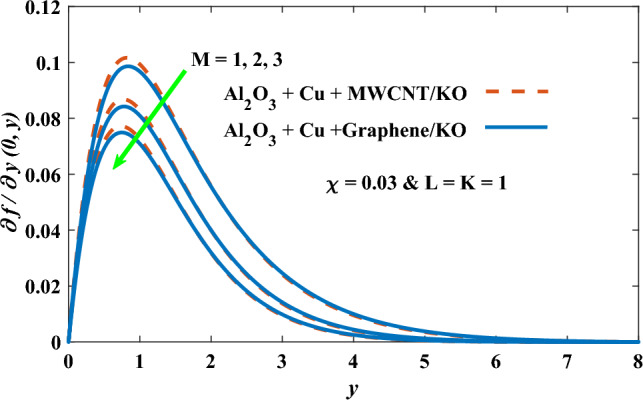
Velocity vs magnetic factor.

**Figure 13:**
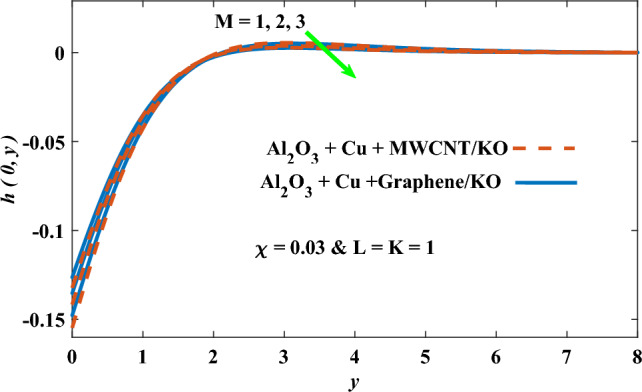
Angular velocity vs magnetic factor.

**Figure 14:**
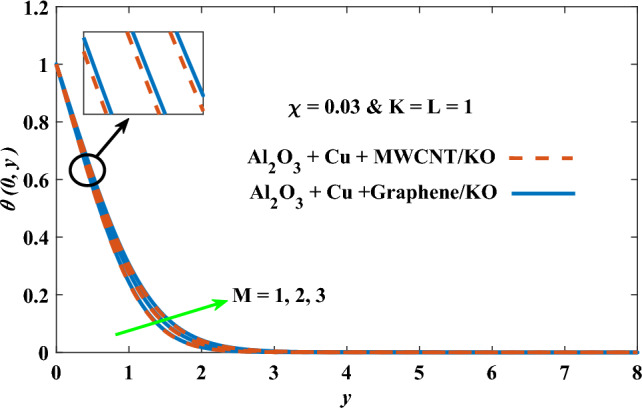
Temperature vs magnetic factor.

**Figure 15:**
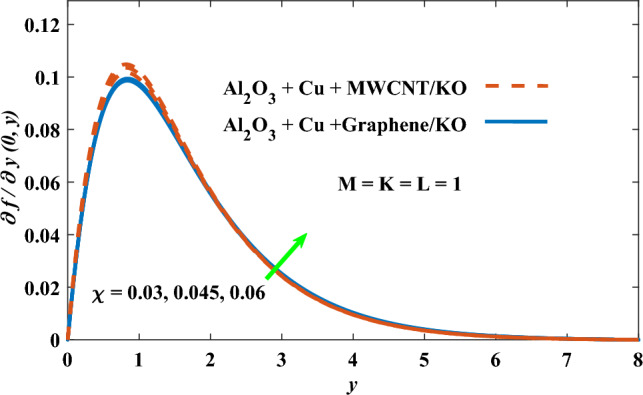
Velocity vs volume fraction factor.

**Figure 16:**
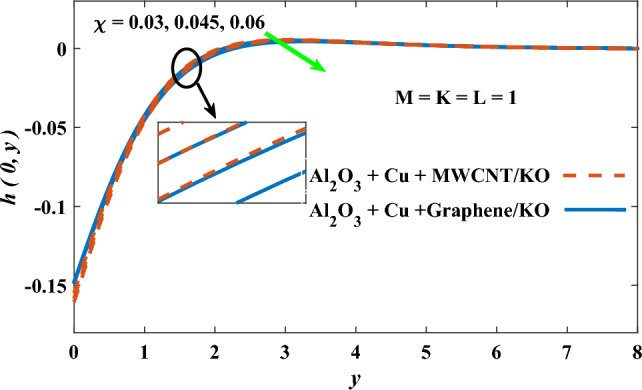
Angular velocity vs volume fraction factor.

**Figure 17:**
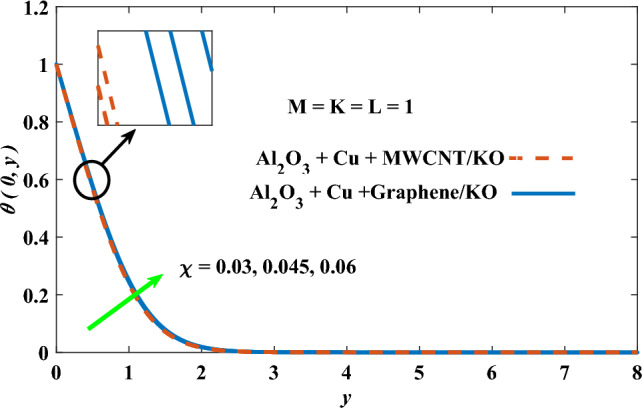
Temperature vs volume fraction factor.

**Figure 18:**
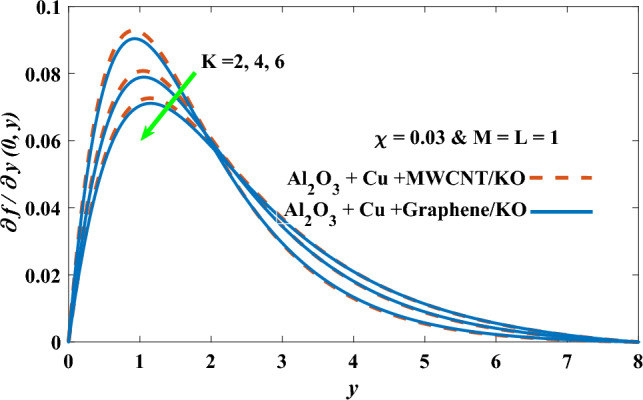
Velocity vs micropolar factor.

**Figure 19:**
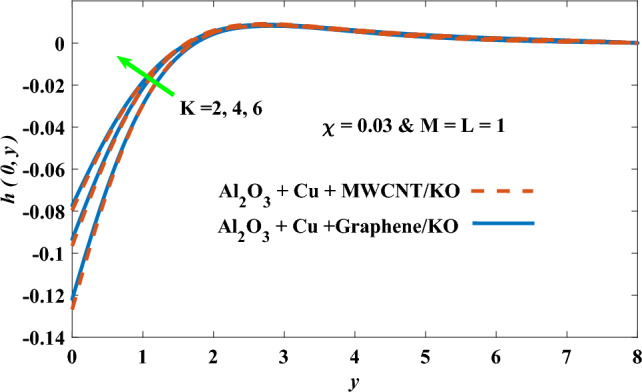
Angular velocity vs micropolar factor.

**Figure 20:**
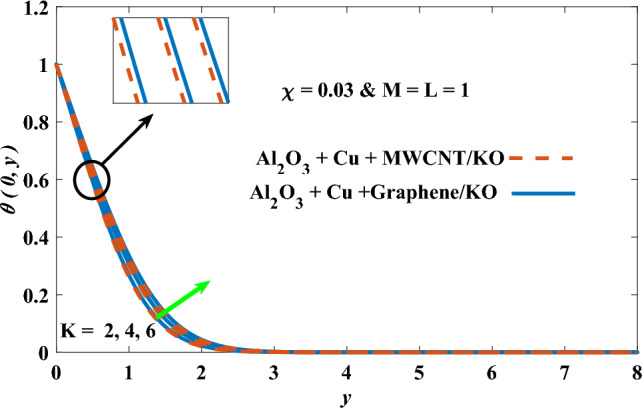
Temperature vs micropolar factor.

**Figure 21:**
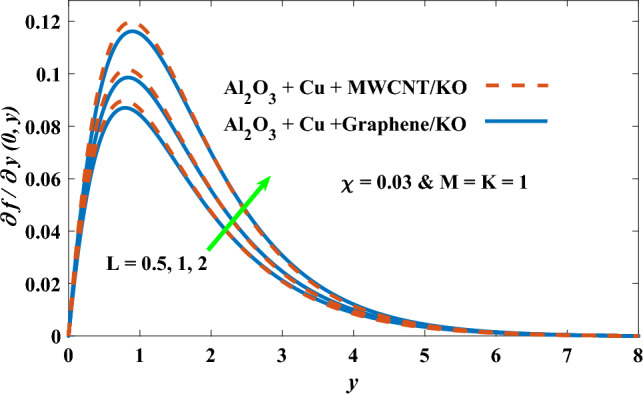
Velocity vs radiation factor.

**Figure 22:**
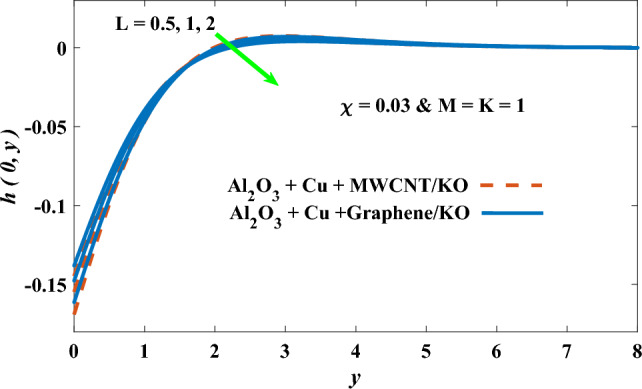
Angular velocity vs radiation factor.

**Figure 23:**
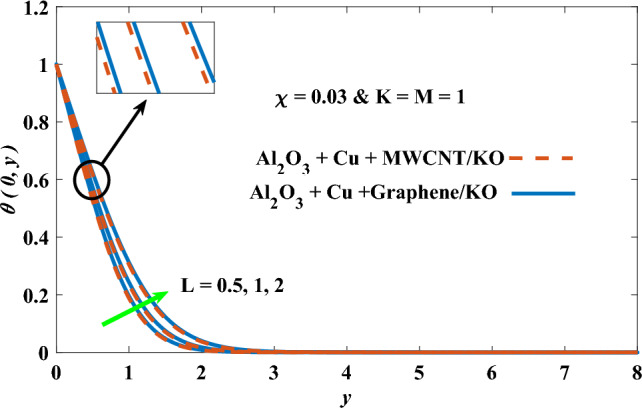
Temperature vs radiation factor.

In this part, the simulation results are graphically presented, elaborated upon, and analyzed in order to offer a comprehensive grasp on the issue. In addition to providing physical explanations for the responses and behaviors of physical groups when affected by the key factors and analyzing their reflections on flow characteristics and energy transport by natural means. Al_2_O_3_ + Cu + Graphene/KO and Al_2_O_3_ + Cu + MWCNT/KO are the used ternary hybrid nanofluids, assuming the graphene nanosolids are shaped like platelets, MWCNT is cylindrical, and the other nanosolids are spherical. Figure 4 describes the influence of augmentation of the volume fraction factor of nanosolids on the Nusselt number. The rise in the $\chi$ factor ameliorates the Nusselt number in response to the remarkable improvement in the thermal conductivity of kerosene oil when the values of this factor are increased. This means the augmentation of the volume fraction factor enhances the convective heat transfer process in the kerosene oil. Likewise, skin friction adopts the same behavior when affected by increasing the volume fraction factor, as shown in Fig. 5. This implies that there is a stronger resistance to the flow of fluid over a surface, indicating a higher drag force or frictional force acting on the fluid. Figure 6 shows a visualization of the relationship between the increase in magnetic field strength and the Nusselt number. Augmentation of the magnetic factor triggers a brake in fluid motion, which is followed by a diminished convective heat transport, and this means that the Nusselt number will minimize. In consideration of the fact that the magnetic factor is inversely related to the motion of fluids, the drag forces experienced by the fluid also diminish, which negatively affects the values of skin friction, which in turn tends to reduce; this behavior is clearly shown in Fig. 7. Figure 8 depicts the extent of the change in the Nusselt number if the micropolar factor values are raised. An increase in the polar factor raises the viscosity of the tri-hybrid nanoliquid, which inhibits its motion and, as a result, reduces its ability to transmit heat. Figure 9 clarifies the opposite response of skin friction caused by elevated micropolar factor values. In situations where the micropolar factor is elevated, the result is a liquid with a higher viscosity, as previously stated. This restricts liquid motion and actually weakens frictional forces. Figures 10 and 11 illustrate how the Nusselt number and drag force depend on the radiation factor. The radiation factor serves as an auxiliary energy source, enhancing the efficacy of both heat transmission and frictional forces. Thereby, it can be indicated that the energy transport and frictional forces of the tri-hybrid polar liquid increase as the amount of emitted thermal radiation increases. The dependence of velocity profiles, angular velocity profiles, and temperature profiles on the magnetic factor is shown in Figs. 12, 13 and 14, respectively. The increment in the magnetic factor means that the magnetic field strength will increase, and this causes a brake in the flow process, or, in other words, it strengthens the resistance of the tri-hybrid liquid’s particles to movement, which will diminish its velocity and angular velocity, while raising its temperature. Figures 15, 16 and 17 are plotted to explore the behaviors of velocity profiles, angular velocity profiles, and temperature profiles under the impact of the volume fraction parameter, respectively. According to the results of the current study and previous studies, elevating the volume fraction values increases the thermal conductivity of the original fluid. This enhances the fluid’s heat-transporting efficiency. Consequently, its velocity and temperature will increase. On the contrary, Fig. 16 reveals a negative relationship between the angular velocity of the original fluid and the volume fraction factor. Figures 18, 19 and 20 visualize the trends of the velocity profiles, angular velocity profiles, and temperature profiles under the influence of the polar factor. By increasing the polar factor values, the temperature and angular velocity contours tend to rise, whereas the velocity contours tend to decline. This generally happens since increasing the polar factor enhances nanofluid viscosity. The positive effect of the thermal radiation factor on velocity and temperature and its negative effect on angular velocity are clearly shown in Figs. 21, 22 and 23. This behavior may be explained by the fact that an increase in the amount of radiation emitted results in adding additional energy sources to the micropolar liquid, which in turn enhances its velocity and temperature.

## Conclusion

Centralizing on filling the research gap by considering the effect of MHD micropolar ternary hybrid nanofluids, the current study considers the nanosolids’ shapes via a mathematical model of the flow of the magnetized micropolar ternary nanoliquid around a spherical shape with thermal radiation effects, which was successfully constructed. On the other hand, the spectral collocation technique (HLSC) has been employed to solve the PDEs and get new numerical outcomes that combine the effects of MHD micropolar ternary hybrid nanofluid parameters that were not studied in the same model. Consequently, we obtained new results that were compared with previous literature and came to an excellent agreement. Moreover, it can contribute to the establishment of future studies based on this study. Depending on that, this study has drawn the following key conclusions:

1. Blade nanosolids give the maximal thermal conductivity ratio, while spherical nanosolids give the minimal ratio.
2. Nanosolids with larger elongations offer kerosene oil the greatest dynamic viscosity ratio.
3. The fluid velocity, frictional forces, and energy transport rate are all suppressed when the micropolar or magnetic factor values rise.
4. As the volume fraction factor values get higher, temperature, velocity, and angular velocity all rise.
5. All examined physical quantities elevate due to the augmentation in radiation factor values.
6. As the volume fraction factor increases, the average percentage improvement in convective heat transfer for Al_2_O_3_ + Cu + MWCNT/kerosene oil compared to Al_2_O_3_ + Cu + graphene/kerosene oil approximately ranges from 0.8 to 2.6%.

Depending on this investigation, there is a lot of future research that can be examined for coming studies. The same problem can be expanded in future work utilizing other mathematical models, such as the Casson model, and it can also develop to comprise ternary hybrid nanofluids with viscous dissipation and Joule heating impacts and incorporated.

## Data Availability

The datasets used and/or analyzed during the current study available from the corresponding author on reasonable request.

## List of symbols

*a*: Radius of the sphere
*B*_*0*_: Magnetic field
*C*_*f*_.: Skin friction coefficient
*G*: Angular velocity
*Gr*: Grashof number
*G*: Gravity vector
*χ*: Volume fraction factor
*M*: Magnetic parameter
*Nu*: Nusselt number
Pr: Prandtl number
*σ*: Electrical conductivity
*x,y*: Dimensional variables along velocity component *z, w*
*N*: Shape factor
*ρc*_*p*_: Heat capacity
*qr*: Rosseland diffusion approximation for radiation
*j*: Micro-inertia density
*r(x)*: The radial distance
*ψ*: Stream function
*T*_*∞*_: Ambient temperature
*z*: Component of velocity
*w*: Component of velocity
*v*_*f*_: Kinematic viscosity
*k*.: Thermal conductivity
*β*: Thermal expansion coefficient
*T*_*w*_: Wall temperature $$(K)$$
*κ*: Vortex viscosity
*µ*: Dynamic viscosity
*ρ*: Density
$T$: Temperature of the fluid
*C*_*1*_*, C*_*2*_: Viscosity coefficient
*ω*: Particle’s sphericity
*ϕ*: Spin gradient viscosity
*σ*, k**: Stefan–Boltzmann, mean absorption coefficients
*θ*: Temperature
*K*: Micropolar parameter
*L*: The thermal radiation parameter

## Subscript

*nf*: Nanoliquid
*f*: Original liquid
*thnf*: Ternary hybrid nanoliquid
*hnf*: Hybrid nanoliquid
